# The Possible Role of *Helicobacter pylori* in Gastric Cancer and Its Management

**DOI:** 10.3389/fonc.2019.00075

**Published:** 2019-02-22

**Authors:** Khalid O. Alfarouk, Adil H. H. Bashir, Ahmed N. Aljarbou, AbdelRahman M. Ramadan, Abdel Khalig Muddathir, Sari T. S. AlHoufie, Abdelhamid Hifny, Gamal O. Elhassan, Muntaser E. Ibrahim, Saad S. Alqahtani, Shakir D. AlSharari, Claudiu T. Supuran, Cyril Rauch, Rosa Angela Cardone, Stephan J. Reshkin, Stefano Fais, Salvador Harguindey

**Affiliations:** ^1^Alfarouk Biomedical Research LLC, Tampa, FL, United States; ^2^Hala Alfarouk Cancer Center, Khartoum, Sudan; ^3^Al-Ghad International College for Applied Medical Sciences, Medina, Saudi Arabia; ^4^American Biosciences, Inc., New York City, NY, United States; ^5^Institute of Endemic Diseases, University of Khartoum, Khartoum, Sudan; ^6^College of Pharmacy, Qassim University, Buraydah, Saudi Arabia; ^7^Al-Ghad International College for Applied Medical Sciences, Jeddah, Saudi Arabia; ^8^College of Dentistry, Taibah University, Medina, Saudi Arabia; ^9^Department of Pharmacognosy, Faculty of Pharmacy, University of Khartoum, Khartoum, Sudan; ^10^Department of Clinical Laboratory Sciences, Faculty of Applied Medical Sciences, Taibah University, Medina, Saudi Arabia; ^11^Al-Azhar University, Cairo, Egypt; ^12^Unaizah College of Pharmacy, Qassim University, Unaizah, Saudi Arabia; ^13^Clinical Pharmacy Department, College of pharmacy, Jazan University, Jazan, Saudi Arabia; ^14^Department of Pharmacology and Toxicology, College of Pharmacy, King Saud University, Riyadh, Saudi Arabia; ^15^Department of Pharmacology and Toxicology, Medical College of Virginia, Virginia Commonwealth University, Richmond, VA, United States; ^16^University of Florence, Florence, Italy; ^17^School of Veterinary Medicine and Science, University of Nottingham, Nottingham, United Kingdom; ^18^Department of Biosciences, Biotechnologies and Biopharmaceutics, University of Bari, Bari, Italy; ^19^Department of Oncology and Molecular Medicine, National Institute of Health, Rome, Italy; ^20^Institute for Clinical Biology and Metabolism, Vitoria, Spain

**Keywords:** *Helicobacter pylori*, gastric cancer, pH, inflammation, pharmacology

## Abstract

*Helicobacter pylori* (HP) is a facultative anaerobic bacterium. HP is a normal flora having immuno-modulating properties. This bacterium is an example of a microorganism inducing gastric cancer. Its carcinogenicity depends on bacteria-host related factors. The proper understanding of the biology of HP inducing gastric cancer offers the potential strategy in the managing of HP rather than eradicating it. In this article, we try to summarize the biology of HP-induced gastric cancer and discuss the current pharmacological approach to treat and prevent its carcinogenicity.

## Historical Background

In 1866, Kussmaul suggested the use of bismuth salts for the treatment of peptic ulcers (1). As bismuth has an oligodynamic effect (toxic to bacteria in a minuscule amount), that was probably the first published evidence of the bacterial role in causing peptic ulcers. After more than 100 years scientists use Bismuth subsalicylate in treating gastritis after conventional antibiotics have failed in improving its symptoms (2). In 1875, Bottcher and Letulle hypothesized that ulcers are caused by bacteria (1). Over a century later Marshal and Warren isolated a spiral bacteria that causes gastritis in 1983 and 1984 (3, 4). After that, the prevailing medical dogma of gastritis was shifted from stress-induced gastritis to be defined as an infectious disease. In 1997, Tomb et al. published the whole genome sequence of that spiral bacterium that was termed by *Campylobacter pylori did* (5) and later became *Helicobacter pylori* (HP) (6).

*Helicobacter pylori* (HP) was identified as a common cause of chronic gastritis (3, 4). Chronic gastritis, perhaps through mucosal pH changes and activation of chemical carcinogens, may lead not only to gastric ulcers but also to stomach cancer and other malignancies of the gastrointestinal tract (7). In this context, we discuss the role of HP in gastric cancer and new possible measures of prevention and treatment.

## Clinical Perspective

HP originated in Africa over 100,000 ago and has become a very useful tool to monitor human migration and detect human ancestry (8–11). More than 50% of the world's population is infected with HP in their stomachs, with a higher prevalence rate in developing countries as compared to developed countries (12). Although <20% of those infected may develop any symptoms, many develop wide-ranging symptomatology. Moreover, HP can be acquired during childhood (13), and its transmission can be associated with childhood episodes of gastroenteritis (13, 14).

HP infection often results in deficiencies in micronutrients such as vitamin A, C, E, Iron, Copper, and B12 (15). Also, HP alters nocturnal melatonin secretion, which might perturb its gastroprotective effects and lead to disturbances of the upper digestive tract (16). Also, HP alters the antioxidant properties of melatonin (17). HP viability may be impaired by ascorbic acid, as its growth in the stomach is increased in patients with low ascorbic acid, while the disappearance of the bacterium increases stomach ascorbic acid (18, 19). Therefore, HP alters the redox status of the organism by scavenging the anti-oxidants from the body.

HP is correlated to many aging-related diseases, and also it increases the susceptibility to other infectious diseases such as cholera (20). HP is also a causative agent of acne vulgaris and Polycystic Ovarian Syndrome (PCO_S_) due to its ability to induce hyperprolactinemia (21). HP also raises blood pressure (22) and increases the risk of ischemic heart disease (23). Its infection may also increase the incidence of diabetes (24), yet evidence suggests that HP eradication may result in weight gain (25). HP has an essential role in preventing diseases, such as asthma (26, 27) or as an immunomodulator against infectious agents such as Mycobacterium tuberculosis (28).

HP has been designated by the WHO as a carcinogen (20, 29) because it can develop: (i) gastric adenocarcinoma and (ii) MALT lymphoma (mucosa-associated lymphoid tissue) (30). However, not every infected individual will develop gastric cancer due to (1) the nature of HP and (2) host vulnerability (31, 32). Surprisingly, HP has also been shown to play a critical role in the prevention of esophageal carcinoma (33). Indeed, in western countries, lowering the prevalence rate of HP has been associated with an increase in the incidence of esophageal adenocarcinomas (14), possibly due to a hygienic culture that excludes naturally occurring defense mechanisms. The eradication of HP not only results in an increased incidence of developing esophageal adenocarcinoma but also appears to decrease its ability to delay or prevent gastric cancer (34–36). However, HP has no relationship with esophageal squamous cell carcinomas (37).

## Helicobacter Pylori-Induced Gastric Cancer

HP expresses a variety of genes involved in its pathogenicity and remodeling of the microenvironment. Here, we review several of these factors that may be involved in HP-mediated carcinogenesis.

### Urease

Urease enzyme plays a critical role in maintaining the HP niche as it hydrolyzes urea into ammonia. This leads to a neutralization of the acidity around the bacteria to create a suitable microhabitat (38). It also facilitates diffusion through mucus by reducing its viscoelasticity (39) and modulates the host's immune response against HP (40, 41). Therefore, urease enzyme is a critical factor that determines HP fitness (42) but not its pathogenesis (43).

Urease catalyzes the breakdown of urea into NH_3_ and CO_2_ (44, 45), which provide both acid-neutralizing and acid-buffering capacities. It appears conceivable that urease is a cytoplasmic enzyme, since the urease activity increases in media where the pH was progressively lowered, without detectable changes of the bacterial cytoplasmic pH, and without evidence of bacterial membrane damage.To support this finding, a transporter has been identified encoded by the ureI gene capable of delivering urea to the cytoplasm (46), where urease enables neutralization and buffering capacities (47). It is now apparent that the activities of the transporter and enzyme are coupled not only functionally but physically as well. Under acidic conditions a neosynthesis of bacterial proteins (e.g., arginase and carbonic anhydrase) has been shown, many of them directly or indirectly involved in pH regulation of both cytosol and cytoplasmic vacuoles. Arginase is involved in providing the substrate to urease to produce L-ornithine and urea (48).

### Carbonic Anhydrase

Carbonic anhydrase (CA) is a form of family of zinc-containing metalloenzymes that catalyze the interconversion of carbon dioxide (CO_2_) and water (H_2_O) to form carbonic acid that dissociates to form bicarbonate (${\text{HCO}}_{3}^{-}$) and Hydrogen ion (H^+^). It is expressed in both prokaryote as well as eukaryote (49), and even within the eukaryotic cells, it is found in many subcellular compartments, such as the cytosol, mitochondria or anchored to membranes (50, 51). CAs are ubiquitously expressed in many tissues of human body that reflects the importance of their physiological functions in maintaining the buffering capacity of the biological systems as well as in biosynthetic processes (52). It is associated with many other diseases including cancer.

Regard the HP, HP has two *forms* of CA, an alpha-type enzyme (HpαCA) and the Hp beta CA (HpβCA) (53, 54). While HpαCA supports urease activity, HpβCA supports bacterial growth at acidic pH (54). Therefore, HpCAs plays crucial role in adapting of HP to gastric environment and supports its fitness.

### Lewis Antigen

HP expresses Lewis antigens on their surface as part of their lipopolysaccharide components (55, 56). The Lewis antigen system is a human blood group system based upon genes on chromosome 19 p13.3 (FUT3 or Lewis gene). HP NCTC11637 expresses a lipopolysaccharide (LPS) that comprises an O-antigen side chain with structural homology to the human blood group antigen Lewis X [Le(x)] (57). Therefore, expression of the Lewis antigen could be for mimicry (anti-predation strategy to avoid immune system). Also, Lewis antigens expressed on the bacterial surface facilitate adherence of HP to gastric epithelial cells (tissue tropism) (58). Therefore, humans carrying blood group A, B are relatively resistant to HP adherence to their epithelium (57, 59). HP strains differ in their expression of Lewis antigen into Le^x^, Le^y^, both, Le^a^, sialyl-Le^x^ or negative for both (60). Le^x^ and Le^y^ are correlated with cagA+ and s1/m1 VacA. Among the western population, the dominant phenotypes are Le^X^ and Le^y^ while Le^a^ and Le^b^ are found in a smaller proportion (61). Possessing of Le^x^ and Le^y^ leads to higher HP internalization rates by gastric epithelium as compared to Le^a^ and Le^b^ or non-expressing Lewis antigen (62).

Lewis antigen and urease are inversely related because urease promotes HP survival and colonization (63) while inhibiting internalization (64). Moreover, we are not sure if the concomitant loss of urease activity occurs due to phenotypic changes from mucus to adherent epithelial phenotype nor if this loss of urease activity and acquiring of Le^x^ and Le^y^ is beneficial for immune evasion (65). Therefore, HP internalization might occur during the development of gastric atrophy as an unfavorable habitat. Adaptation in hostile habitats that affect organismal multiplication is called “cost of adaptation.” Therefore, the acquisition of Lewis antigen is associated with the cost of adaptation. In other words, acquiring of Lewis antigen will support HP' survival, but it affects negatively on the proliferation rate.

### VacA

VacA (vacuolating cytotoxin) is a secreted protein encoded by the vacA gene. All HP strains have the vacA gene, but they differ in their expressivity (66). VacA secretion is more frequent in patients with gastric cancer as compared to patients with gastritis *(alone)* (67), indicating a connection between expressivity and pathogenicity. Characterization of 59 different HP isolates revealed the existence of three distinct families of vacA sequences (s1a, s1b, and s2) and two separate families of middle-region alleles (m1 and m2) (68). The sub-strain that has the m1/s1 allele is the most virulent isoform regarding its ability to induce inflammation (69). VacA is composed of the P55 and P33 proteins. P55 is responsible for producing pores within the gastric epithelium while P33 disrupts mitochondrial fission machinery upon its inoculation (70), inducing cellular death of the epithelium (71). VacA also alters the maturation and trafficking of lysosomal enzymes (72). Also, VacA inhibits T-cells population expansion (73), and so it may promote survival of various phenotypes.

### CagA

CagA is a 40 kbp horizontally acquired gene (6, 74) that encodes for the Type IV Protein Secretion System (T4SS). T4SS delivers cagA-oncoprotein (75–77) to suppress apoptosis (78). Outer membrane proteins (OMPs) and cagA target mitochondria. In this regard, development of gastric cancer due to mitochondrial injury is compatible, or at least parallel, with what Otto Warburg had earlier hypothesized on the respiratory impairment of cancer cells (79).

HP is heterogeneous in inducing pathogenesis (12). If cagA^+^ is associated with developing gastric adenocarcinoma, either cagA^+^ or cagA^−^ can induce β-Lymphoma (30). The growth of MALTomas (MALT-NHL) is a more immune-dependent growth. It is clear that HP interacts with the immune system with a “gold panning” strategy to increase IL-2 expression through T-cells (80). In summary, eradication of HP might represent a potential strategy for treating these tumors (81), either low-grade β-cell lymphoma and to some extent higher grades of this tumor (82–84).

CagA is associated with a higher production rate of cytokines (85, 86). HP seropositivity has been classified to be either cagA^+^ or cagA^−^ sub-strains. The presence of cagA^+^ modulates epithelial activity that acts as a phosphatase enzyme (dephosphorylation) resulting in pro-inflammation by releasing IL-8 (87), MAPK (88), and NF-KB (89–91). Hence, cagA^+^ might be considered to be a hallmark of HP carcinogenicity. Some studies have shown that the cagA^+^ strain significantly increases epithelial proliferation rate either directly (92, 93) or through the induction of hypergastrinemia (increased gastrin level) (94, 95) while another study shows that cagA+ apoptosis indices are increased (96, 97). Such contradictions might reflect that epithelial proliferation and apoptosis are contagious processes that are controlled by the cagA+ strain in a way that expresses a multistage progression of epithelial transformation.

### Outer Proteins (BabA2)

The outer membrane protein (OMP) of HP, babA2, is associated with an increased risk of gastric cancer. BabA2 is a member of a family of highly conserved OMP, is encoded by strain-specific gene *babA2* and binds the Lewis^b^ (Le ^b^). BabA2 is commonly found in phenotypes that adhere to the epithelium (14, 98). The BabA2+ sub-strain in Le^b^ expressing mice results in developing atrophy anti-parietal antibodies, i.e., the development of gastric atrophy is considered an autoimmune disease (98–100) and so results in chronic inflammation. The combination in a triple positive strain of the above proteins (BabA2, CagA+, and VacA) is a reliable indicator of the possibility of inducing carcinogenicity (101).

In conclusion, if urease is responsible for establishing a suitable platform for HP colonization, Lewis antigen is an essential protein that serves HP fitness under unfavorable habitat conditions followed by CagA, Bab, and VacA that makes HP occupy the gastric epithelium and induce inflammation. In summary, all these genes and their subsequent proteins are working orchestrally in a cascade manner to serve the evolutionary trajectory of HP.

## Human Response

Some of the keys elements that interact with HP to induce gastric cancer include.

### β–Catenin

Beta-catenin (β-catenin) is a protein encoded by CTNNB1 gene on band p12 placed on the short arm of chromosome 3, a region that is affected by a somatic alteration in the tumor (102). β-catenin is a protein that plays a paramount role in the coordination of both cell-cell adhesion and gene transcription.

β-catenin is a proto-oncogene that has been found to accumulate inside the nucleus in precancerous lesions of gastric cancer (103). β-catenin is associated with several types of tumors including primary hepatocellular carcinoma, ovarian carcinoma, breast cancer, lung cancer, colorectal cancer, basal cell carcinoma, prostate cancer, pilomatrixoma, medulloblastoma, Head and neck squamous cell carcinoma and glioblastoma (104–108).

HP activates β-catenin expression (76, 109, 110) such that β-catenin activates its expression via HP as a positive feedback mechanism to induce intestinal metaplasia. Initially, intestinal metaplasia is dependent on cagA (111) and remains latently independent even after HP eradication (112).

### EGFR

EGFR is a member of the ErB family which is structurally related to tyrosine kinase receptors Her1 (EGFR or ErB-1), Her2 (ErB-2), Her3 (ErB-3), and Her4 (ErB-4) (113). Epidermal growth factor receptors (EGFR) are cell surface expressed proteins. EGFR is a target for Epidermal Growth Factor (EGF) and Transforming Growth Factor–alpha (TGF-alpha) ligands to stimulate cellular proliferation. EGFR is expressed in several carcinomas and induces cellular transformation (114). Those carcinomas are EGFR-dependent in their survival and growth (114).

HP keeps in balance an epithelial proliferation/apoptosis ratio (96, 115, 116). HP increases such ratio via activation of EGFR (117, 118) i.e., HP increases cellular proliferation through activation of EGFR.

Targeting EGFR, perhaps as a too potent strategy for prevention of HP-inducing gastric cancer; due to its ability to induce excessive apoptosis and/or “oncogenic shock” (113, 119).

## Inflammation as a Result of Helicobacter Pylori—Human Interactions

The correlation between gastric inflammation and ulcers, gastritis, and gastric cancer had been first explored by Stahl in 1728 and Nevpeu as soon as 1821 (1) and later on by many other groups [for a review see: Harguindey, (7)]. HP is a chronic infection that leads to chronic inflammation (41, 120) and so promotes tumorigenesis (gastric cancer) (121, 122). The inflammatory response induced by HP leads to the release of mutagenic substances e.g., metabolites of inducible nitric oxide synthase (iNOS). Nitric oxide can result in a change in reactive nitrogen species that are found in DNA, proteins, etc. (14). Therefore, HP releases free radicals and removes antioxidant agents.

### Phospholipase A_2_ (PLA_2_)

**Figure 1:**
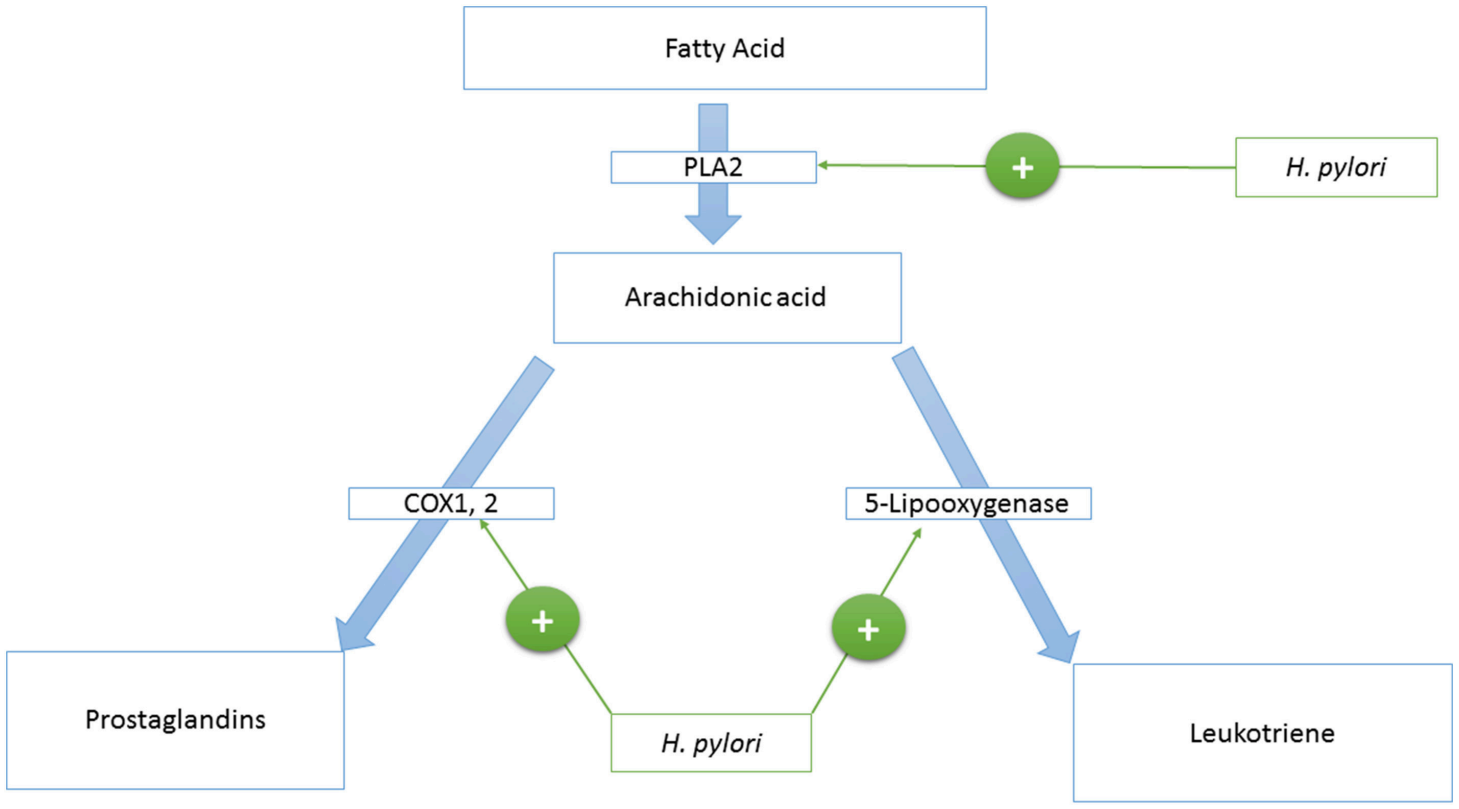
A model that represents the *H. pylori*—host’s eicosanoids interactions.

Phospholipase A2 is an enzyme that catalyzes the production of arachidonic acid from fatty acids. Arachidonic acid is further converted to prostaglandins and Leukotriene by cyclooxygenase and lipoxygenases enzymes, respectively. HP activates the Phospholipase A_2_ enzyme (PLA_2_) (123, 124) (see Figure 1).

### Cyclooxygenase Enzyme 1 and 2 (COX-1,−2)

Cyclooxygenase 1, and 2 (COX-1,−2) are enzymes that catalyze the conversion of prostanoid to prostaglandins. Prostanoid synthesis is thought to be cytoprotective to the stomach and increases production of the pro-aggregatory prostanoid, thromboxane, by platelets (125) (see Figure 1). Alternatively, COX-2 induction by TNF-alpha, INF-gamma, and IL-1 (126–131) is associated with colorectal cancer (132), and HP activates PLA2 and TNF-alpha expression (133–135). Also, COX-2 is present in the atrophic area and malignant gastric lesions (125, 136). Overexpression of COX-2 prevents apoptosis (137). In this way, COX-2 might support the tumorigenic potential of HP.

### Leukotriene

Leukotriene is produced from arachidonic acid by the activity of the 5-Lipooxygenase enzyme, and this is accompanied by the release of histamine (see Figure 1). Leukotriene is associated with gastritis (138, 139), and its receptors have been found to be overexpressed in gastric cancer (140). Leukotriene receptors are persistent in the epithelium of the stomach even after eradication of HP (141).

In summary, HP is related to eicosanoids at multiple levels to create a sustainable chronic inflammatory environment that leads to initiation, development, and progression of gastric cancer.

## Acid-Base Considerations in Etiopathogenesis and Therapy

Last but not least, IL-1β is a TH1 cytokine having a strong acid production inhibitory property (14, 142). Thus, it is not surprising that IL-1β has a pivotal role in initiating the development of gastric adenocarcinomas (7, 143) since inhibition of acid production is a significant step in developing gastric cancer (144). Together with cagA^+^, IL-1β overexpression, it also increases the prevalence of gastric adenocarcinomas (14). TNF-α also inhibits gastric acid secretion (145), and this is also correlated with gastric cancer (146). All these situations can be inserted within the concept of pH-direct and pH-indirect gastrointestinal oncogenesis [for a review, see Harguindey, (7); Table 1].

**Table 1:** Shows some factors that govern *H. pylori*—human (host) that mediates carcinogenesis.

***H. pylori* related factors**	**Human related factors**
Urease	Beta-catenin
Carbonic Anhydrase	
VacA	
CagA	EGFR
Lewis Antigen	
BabA2	

## Notes in the Management of HP Infection

Antibiotics represent the backbone of the currently used strategy of triple therapy. This “triple therapy” strategy consists of two antibiotics (Clarithromycin and Amoxicillin) for HP eradication plus an acid-suppressing agent, usually a proton pump inhibitor to alter the bacterial microenvironment and reduce gastric pain.

**Antibacterial** administration not only leads to post-elimination complications [e.g., the development of esophageal adenocarcinoma, of asthma, of metabolic disorders, etc. (147)]. Furthermore, the efficacy of antibiotics are questionable due to (i) uncertainty of antibiotic ability to create stomach sterility (38), (ii) prevention of *HP'*s tumorigenicity (120), or even (iii) induction cost of resistance rather than diminishing microbial-host co-evolution (i.e., perturbation of ecological interactions) (25, 148).

- **Clarithromycin** is a semi-synthetic macrolide antibiotic derived from erythromycin which at the same time is originated from *Streptomyces erythreus* (112, 149). It binds to the part of the ribosome (50s ribosomal subunit), and so it affects peptide translation. In addition to its bactericidal activity, Clarithromycin inhibits the production of superoxide that is released from neutrophils and other white blood cells (150). Clarithromycin has a membrane stabilizer property and, so, inhibits the release of pro-inflammatory mediators (151). Clarithromycin inhibits IL-8 production through affecting AP-1 and NF-kappa B expression. So, it is useful in treating HP infection (152, 153). That is why Clarithromycin represents the core of the three-pronged therapy approach. However, if Clarithromycin failed, Levofloxacin could be used.
- **Levofloxacin** is a suggestive substitue for Clarithromycin resistant strains (154, 155), although Levofloxacin might induce localized inflammation in the form of tendinitis or tendon rupture (156–158).
- **Amoxicillin** is a semi-synthetic Beta-Lactam antibiotic derived from *Penicillium notatum*. It inhibits bacterial cell wall synthesis. Amoxicillin has a high therapeutic index (high safety profile), and it can be combined with Clavulanic acid if some strains develop Beta-lactamase enzymatic inhibition. Metronidazole is a substitute in the case of penicillin hypersensitivity.
- **Metronidazole** is a nitroimidazole compound that acts against anaerobic bacteria and protozoa. Metronidazole is a prodrug activated by an unusual enzymatic system, the Pyruvate: Ferredoxin oxidoreductase (PFO) found in hydrogenosome (an anaerobic version of mitochondrion) (159). PFO leads to activation of Metronidazole, and the product of this reaction leads to the destruction of the helical structure of the DNA of the microorganism.
- **Acid-suppressive agents:** In the past, clinicians used H_2_-receptor antagonists, e.g., Cimetidine, which was later substituted by Famotidine because of drug side effects of the former. Currently, using proton pump inhibitors (PPIs) have become most popular. Moreover, it has also been shown that omeprazole and its analogs may behave as Helicobacter pylori urease inhibitors (160). Two very recent reports add much to the use of proton pumps inhibitors in HP eradication. One shows that the genotypic polymorphisms of HP may be predictive of the of the optimum PPI dose to improve the therapeutic outcome (161). The other highly support the use of proton pump inhibitors in the HP-mediated gastric atrophy eradication approaches (162). However, it appears clear actually that the use of PPI should be mandatory in the prevention of gastric cancer relapses after endoscopic submucosal dissection for early gastric cancer (163), and this is of course of paramount importance. A role of pH and proton pumps for the growth of a variety of infectious agents has been provided, and it is still under a challenging debate (164). However, it is impressive how the involvement of proton pumps exert a central role a specificity in infectivity of so many microbes and parasites, in a way that proton pump inhibition has proven to induce an apparent anti-infective effect. This has been shown in many bacteria such as of course M. Tuberculosis, where the PPI lansoprazole seems to exert its action through a specific target to cytochrome bc1 (165); but also Salmonella enteric infection, where Omeprazole interferes with virulence and inflammation in infected cells (166); in experimental *Clostridium difficile* infection in mice (167). Some research on new compounds based on biologically active peptide against proton pumps seems to generically support these data (168). Moreover, PPI has been shown to be effective against a series of protozoa, yeasts, and amoebas, including *Giardia lamblia* (169), *Trichomonas vaginalis* (170), *Plasmodium falciparum* (171). The use of appears promising in yeasts (172), and *Dictyostelium discoideum* (173, 174) infections, where a role of proton pumps in intracellular replication has been shown. Therefore, PPIs have a direct bactericidal activity (175–179). This represented an additional antibiotic-like measure within the triple therapy strategy.
- This suppression is beneficial for gastric atrophy. Therefore, in this respect, PPIs might be equivalent to IL-1β and TNF-α. So, it will be wise if someone raises that PPIs perhaps could support gastric atrophy and hypochlorida (180). However, this is not a case, because PPIs show a promising potential effect in treating cancer (181–184).

Recently, the concept of quadruple therapy has also been introduced to the development of drug resistance (185), which reflects the unusual ability of *HP* to adapt.

Administering of NSAIDs as COX-inhibitors is considered as a potential strategy for preventing the development of gastric cancer because they decrease and attenuate the inflammatory environments and so prevent and/or delay tumor progression. However, cyclooxygenase inhibition will prevent mucous formation that leads to a decrease of the M/A ratio and so increases adherence phenotypes that result in malignant transformed consequences. That is why the administration of NSAIDs to prevent gastric cancer becomes questionable. Part of this idea has been considered previously (186). More comments on anti-inflammatory drugs include:

1. Prevention of PGE-synthesis might alter population dynamics through suppression of M phenotypes as well as alters the entire population by its bactericidal activity (97, 187–190).
2. *HP* infection potentiates Aspirin-induced gastric injury (191).
3. Administration of Aspirin results in increasing of Leukotriene production (192). In this regard, the role of Aspirin in preventing carcinogenesis becomes questionable unless certain tumors rely on PGs rather than Leukotriene.
4. Most probably, administration of steroids will be beneficial due to (i) it decreases incidence of inflammation and so becomes more similar to malaria-selection for populations (193), (ii) the fact that steroids (immunosuppressive therapy) in mice does not alter *HP* colonization (194, 195) and so it is superior to NSAIDs, (iii) steroid as immunosuppressant is beneficial in delaying and/or preventing gastric atrophy, and finally, (iv) HP elicits and activates Phospholipase-A2; where PL-A2 is a critical player in producing the inflammatory mediators, e.g., Prostaglandins, Leukotrienes, etc. (123, 124). Interestingly, down-regulation of inflammatory response might, represent a potential preventive strategy for developing gastric cancer. However, chronic administration of steroids might lead to bone marrow suppression and adrenal atrophy.

Rendering HP to acquire the cagA Island, and/or reversing the *HP* population to become cagA- might represent potential preventive strategies against *HP* induction of gastric adenocarcinoma as well as serving a protective role against developing esophageal adenocarcinoma and other diseases.

## Does HP Behave Like a Cancer Cell?

Tissue acidification is a framework of activities from the internal compartments of the cells to the extracellular microenvironment. The cellular events include the multifunction cascade of internal vesicles, from endosome to phagosome, but this process is highly dependent on the cytosolic pH, that has to be considered a key factor is dictating the vesicle fission and/or fusion. We know for instance that the lipid composition of both the internal and extracellular vesicles may change depending on the pH, and this is particularly true under tumor condition (196). The nature and function of acidic vesicles markedly change dependently on the pH condition, thus conditioning the level of maturation of the vesicles themselves. From a mechanistic point of view the role of proton pumps is critical in orchestrating the pH control in both the intracellular and extracellular microenvironment, and this, in turn, has a crucial role in regulating both the intracellular pathway following membrane receptors triggering (e.g., apoptotic pathways) and the activation of a series of enzymatic cascades, such as caspases. Between the proton pumps, the V-ATPase seem to exert a central role as pH sensors (197). V-ATPase has a crucial involvement in maintaining the intracellular pH gradients in normal condition, through a continuous H^+^ transferring from the cytosol to the acidic vesicles. This activity, on the one hand, avoids acidification of the cytosol, on the other hand, maintain an acidic pH within the internal vacuoles (198). This background may explain a mechanism that pathogenic bacteria use to evade internal killing through alterations in pH homeostasis. The pathogenetic mechanism of HP, in fact, include a pH modulator such as urease. However, to date, it is not entirely clear whether the acidification derangement is aimed at targeting the pH regulation of the bacterium itself or the pH of the gastric environment where the bacterium lives. By analogy with the M. tuberculosis, we know that the normal phagosomal acidification fails to develop because the bacterium interferes with internal vesicles maturation. However, we don't focus enough on both the mycobacterial effectors that inhibit vacuoles maturation and the real mechanism of action.

### Similarities Between HP and Cancer Cells

This, on the one hand, supports the use of PPI as a new class of anti-infectious agents without a clear antibiotic activity, on the other hand, raises the interesting hypothesis that microbial agents may behave like tumor cells in their ability to select cells able to survive in the acidic microenvironment. Some analogies between unicellular microorganisms and malignant tumors cells have been provided in the last decade. The most striking evidence of this amoeba-like behavior of cancer cells came from the way tumor cells feed on other cells when starved or placed in low nutrient supply conditions. This was called tumor cannibalism (199, 200). Moreover, a protein and a gene in common between cancer cells and amoebas have been described (TM9SF4) and recalled Tumor Cannibalism Associated Protein 1 (TUCAP-1) (201). That behavior was very similar to the one used by unicellular microorganisms against bacteria (202). Intriguingly, TUCAP-1 has been shown to be involved in tumor acidification by modulating the activity of proton pumps, such as V-ATPases (203). All in all these results led to hypothesize that cancer cells came back to a very primitive condition (atavistic state) where the cells aimed to survive against the other living beings (204).

Moreover, it has been shown as proton pump inhibitors exert a particular antibacterial activity against HP *in vitro*, and it was clearly pH dependent. In fact, the bactericidal activity specifically against both resting (in the buffer) and growing (in broth) HP was significantly higher at pH 5 as compared to that at pH 7. On the other hand, we know that not only cancer neoplasms are acidic (205) but that both cancer cells and tumors are extremely sensitive to proton pump inhibitors, at pre-clinical and clinical levels (181), and there is a list of proton exchangers inhibitors that have been proven highly effective against cancer (198).

All in all, there are many analogies in general between microbes and cancer cells, but most of all between HP and cancer cells. A question, however, remains open actually. Does HP behave like a cancer cell? Or does the cancer cell that indeed acts the HP?

## Conclusion

HP could be seen as a naturally occurring, “acquired normal flora” and immunomodulating bacterium. It is evolutionarily designed for co-existence with rather than extinction of other bacterial flora. HP induced inflammation might, under certain circumstances, stimulate Prostaglandin E2 (PGE-2) which restores the mucous membrane of the stomach to normal homeostasis. This habitat restoration re-populates the mucous adherent phenotype (M) and so re-structures levels of M phenotypes into the already existing epithelial adherent phenotypes (A) In other words, the presence of mucus helps to maintain HP diversity as well as to stabilize the population size of HP in the stomach. Perhaps the presence of mucus can represent a proxy for the M-phenotype distribution as it relates to (i) the provision of L-lactic acid as a growth enhancer (206) and (ii) how HP obtains benefits from the mucosal flora which is necessary for its survival (207). Further, the normal epithelium is a suitable habitat for the A phenotype (208) instead of an epithelium that is undergoing intestinal metaplasia (fragmentized habitat).

Chronic inflammation results in the creation of unfavorable habitat, by altering pH, around normal cells which instigated on their malignant transformation (209). This environmental change leads to (i) selection and/or elicits phenotypic plasticity of human cells to become acidophilic phenotypes (i.e., the “cave fish's principle”) (210) and (ii) promotion of transformed phenotypes' fitness using spite strategy (211). However, HP suppresses acid production. Therefore, could cancer be considered a strategic defense against HP? In other words, could cancer be represented as an aggressive immune response against HP? We believe that it is time to change the monolithic model that misrepresents HP as an inducer of gastric carcinogenesis as a purpose.

## Author Contributions

All authors listed have made a substantial, direct and intellectual contribution to the work, and approved it for publication.

### Conflict of Interest Statement

The authors declare that the research was conducted in the absence of any commercial or financial relationships that could be construed as a potential conflict of interest.
